# *catena*-Poly[[[tetra­aqua­neodymium(III)]-di-μ-isonicotinato] chloride]

**DOI:** 10.1107/S1600536811055619

**Published:** 2012-01-18

**Authors:** Jun-Hui Xue, Xiao-Hui Hua, Chun-Ping Li, Yi-Zhuang Xu, Jin-Guang Wu

**Affiliations:** a Chemical Engineering College, Inner Mongolia University of Technology, People’s Republic of China; b The State Key Laboratory of Rare Earth Materials Chemistry and Applications, College of Chemistry and Molecular Engineering, Peking University, People’s Republic of China

## Abstract

In the title complex, {[Nd(C_6_H_4_NO_2_)_2_(H_2_O)_4_]Cl}_*n*_, the Nd^III^ cation is located on a twofold rotation axis and coordinated by four isonicotiniate anions and four water mol­ecules in a distorted square-anti­prismatic geometry. The carboxyl­ate groups of the isonicotinate anions bridge the Nd^III^ cations, forming polymeric chains running along the *c* axis. The Cl^−^ anion is located on a twofold rotation axis and is linked to the polymeric chains *via* O—H⋯Cl hydrogen bonding. Inter­molecular O—H⋯O and O—H⋯N hydrogen bonds are also present in the crystal structure.

## Related literature

For some crystal structures of related lanthanide-isonicotinic acid complexes, see: Chen & Fukuzumi (2009 ▶); Ma *et al.* (1999 ▶); Han *et al.* (2010 ▶); Kay *et al.* (1972 ▶); Duan *et al.* (2010 ▶); Jia *et al.* (2008 ▶); Cheng *et al.* (2007 ▶); Liu *et al.* (2006 ▶); Chai *et al.* (2010 ▶).
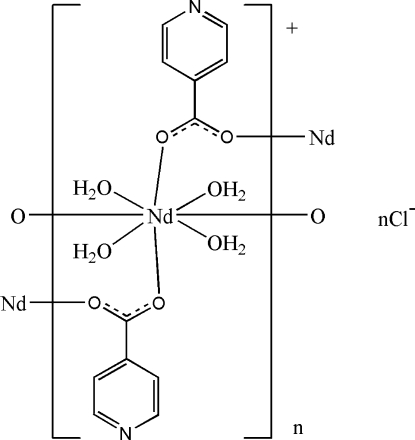


## Experimental

### 

#### Crystal data

[Nd(C_6_H_4_NO_2_)_2_(H_2_O)_4_]Cl
*M* *_r_* = 495.96
Orthorhombic, 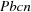

*a* = 8.9223 (18) Å
*b* = 19.684 (4) Å
*c* = 10.151 (2) Å
*V* = 1782.9 (6) Å^3^
*Z* = 4
Mo *K*α radiation
μ = 3.10 mm^−1^
*T* = 173 K
0.18 × 0.17 × 0.15 mm

#### Data collection

Rigaku Saturn724+ CCD diffractometer
Absorption correction: multi-scan (*CrystalClear*; Rigaku, 2007 ▶) *T*_min_ = 0.49, *T*_max_ = 0.63
9085 measured reflections
2056 independent reflections
1764 reflections with *I* > 2σ(*I*)
*R*_int_ = 0.071

#### Refinement

*R*[*F* ^2^ > 2σ(*F* ^2^)] = 0.063
*wR*(*F* ^2^) = 0.190
*S* = 1.33
2056 reflections
122 parameters
6 restraints
H atoms treated by a mixture of independent and constrained refinement
Δρ_max_ = 1.12 e Å^−3^
Δρ_min_ = −1.06 e Å^−3^

### 

Data collection: *CrystalClear* (Rigaku, 2007 ▶); cell refinement: *CrystalClear*; data reduction: *CrystalClear*; program(s) used to solve structure: *SHELXTL* (Sheldrick, 2008 ▶); program(s) used to refine structure: *SHELXTL*; molecular graphics: *SHELXTL*; software used to prepare material for publication: *SHELXTL*.

## Supplementary Material

Crystal structure: contains datablock(s) global, I. DOI: 10.1107/S1600536811055619/xu5361sup1.cif

Structure factors: contains datablock(s) I. DOI: 10.1107/S1600536811055619/xu5361Isup3.hkl

Supplementary material file. DOI: 10.1107/S1600536811055619/xu5361Isup5.cdx

Additional supplementary materials:  crystallographic information; 3D view; checkCIF report

## Figures and Tables

**Table 1:** Selected bond lengths (Å)

Nd1—O1^i^	2.425 (6)
Nd1—O2	2.385 (5)
Nd1—O3	2.544 (6)
Nd1—O4	2.480 (6)

Symmetry code: (i) 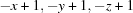.

**Table 2:** Hydrogen-bond geometry (Å, °)

*D*—H⋯*A*	*D*—H	H⋯*A*	*D*⋯*A*	*D*—H⋯*A*
O3—H6⋯Cl2^ii^	0.95 (6)	2.29 (6)	3.211 (7)	163 (6)
O3—H7⋯N1^iii^	0.97 (4)	1.72 (4)	2.685 (10)	174 (9)
O4—H8⋯Cl2^i^	0.96 (4)	2.15 (5)	3.041 (6)	155 (6)
O4—H9⋯O3^iv^	0.95 (2)	1.93 (3)	2.841 (8)	160 (8)

Symmetry codes: (i) 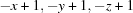; (ii) 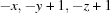; (iii) 
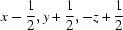
; (iv) 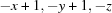.

**Fig. 1.**
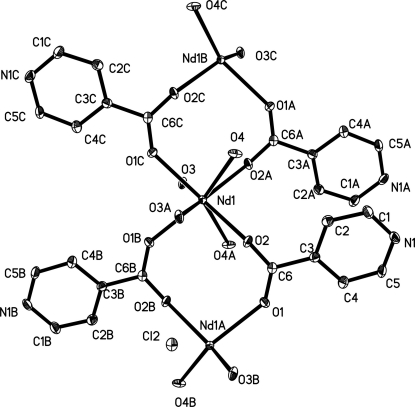
The molecular structure of the title compound, displacement ellipsoids drawn at 30% probability level. The Hydrogen atoms have been omitted for clarity.

## supplementary crystallographic information

### Comment

In the synthesis of prepare a lanthanide-isonicotinamide complex, the isonicotinamide changed to isonicotinic acid, resulting in the title complex.

Among aromatic carboxylic acids, isonicotinic acid has a conjugated structural motif and can be used to construct extended structures because it is unsymmetrical divergent ligand. Isonicotinic acid with metal ions may have various coordination modes (Chen *et al.*, 2009). The molecular structure of the title compound is shown in Fig. 1. Nd(III) is 8-coordinated to four oxygen atoms from four isonicotinic acid molecules and four water molecules. Each ligand is coordinated to two metal ions as bidentate ligands via two oxygen atoms of carboxyl group. One metal ion is connected to four ligands, thus an extensive network form. Nd(III) is located at the center of a slightly distorted square antiprism. Nd-O distances are from 2.385 to 2.544 Å. The mean Nd-O bond lengths, 2.4585 Å is larger than the mean Sm-O bond lengths (2.426 Å), which is consistent with the lanthanide contraction. The O—H···Cl, O—H···O and O—H···N hydrogen bonds form a three-dimensional network.

For lanthanide-isonicotinate complexes, several structures have been observed. For example, Ln(III) ions can coordinate to six carboxylate oxygen atoms of bridging isonicotinate groups and to two water molecules (Ma *et al.*, 1999); or may coordinate to four carboxylate oxygen atoms of bridging isonicotinate groups, two carboxylate oxygen atoms of the chelating isonicotinate group, and two water molecules (Ma *et al.*, 1999; Han *et al.*, 2010; Kay *et al.*, 1972); or have structure similar to the NdCl-isonicotinic acid complex reported here (Chen *et al.*, 2009). Because Cl^-^ or NO_3_^-^ does not coordinate to lanthanide ions, so Nd(III) chloride or nitrate ion-isonicotinic acid complexes have the similar coordination sphere (Duan *et al.*, 2010; Jia *et al.*, 2008). When oxalate ligands, chromate ions or other ligands are involved, the coordination situations are a little different (Cheng *et al.*, 2007; Liu *et al.*, 2006; Chai *et al.*, 2010) because oxalate ligand, chromate ions or other ligands also coordinate to metal ions.

### Experimental

NdCl_3_ (1 mmol) and isonicotinamide (3 mmol) were dissolved in 3ml water and 6 ml ethanol. The solution was put on a water bath, temperature was raised to 80°C. Small aliquots of EtOH were periodically added to the solution during the heating process to prolong the reaction time. The resulting mixtures were filtered and left for crystallization in room temperature, the suitable crystals for X-ray diffraction measuraments were obtained in two weeks.

### Refinement

The C-bound H-atoms were placed in calculated positions (C—H 0.930 Å) and were included in the refinement in the riding model approximation, U_iso_(H) = 1.2U_eq_(C). The O-bound H atoms were located in a difference Fourier map and were refined with U_iso_(H) = 1.2U_eq_(O).

### Crystal data

**Table:**

[Nd(C_6_H_4_NO_2_)_2_(H_2_O)_4_]Cl	*F*(000) = 972
*M**_r_* = 495.96	*D*_x_ = 1.848 Mg m^−^^3^
Orthorhombic, *P**b**c**n*	Mo *K*α radiation, λ = 0.71073 Å
Hall symbol: -P 2n 2ab	Cell parameters from 4581 reflections
*a* = 8.9223 (18) Å	θ = 2.0–27.5°
*b* = 19.684 (4) Å	µ = 3.10 mm^−^^1^
*c* = 10.151 (2) Å	*T* = 173 K
*V* = 1782.9 (6) Å^3^	Block, blue
*Z* = 4	0.18 × 0.17 × 0.15 mm

### Data collection

**Table:**

Rigaku Saturn724+ CCD diffractometer	2056 independent reflections
Radiation source: fine-focus sealed tube	1764 reflections with *I* > 2σ(*I*)
graphite	*R*_int_ = 0.071
Detector resolution: 28.5714 pixels mm^-1^	θ_max_ = 27.5°, θ_min_ = 2.1°
ω scans at fixed χ = 45°	*h* = −11→6
Absorption correction: multi-scan (*CrystalClear*; Rigaku, 2007)	*k* = −25→23
*T*_min_ = 0.49, *T*_max_ = 0.63	*l* = −12→13
9085 measured reflections	

### Refinement

**Table:**

Refinement on *F*^2^	Primary atom site location: structure-invariant direct methods
Least-squares matrix: full	Secondary atom site location: difference Fourier map
*R*[*F*^2^ > 2σ(*F*^2^)] = 0.063	Hydrogen site location: inferred from neighbouring sites
*wR*(*F*^2^) = 0.190	H atoms treated by a mixture of independent and constrained refinement
*S* = 1.33	*w* = 1/[σ^2^(*F*_o_^2^) + (0.0763*P*)^2^ + 8.5206*P*] where *P* = (*F*_o_^2^ + 2*F*_c_^2^)/3
2056 reflections	(Δ/σ)_max_ = 0.004
122 parameters	Δρ_max_ = 1.12 e Å^−^^3^
6 restraints	Δρ_min_ = −1.06 e Å^−^^3^

### Special details

**Table:**

Geometry. All esds (except the esd in the dihedral angle between two l.s. planes) are estimated using the full covariance matrix. The cell esds are taken into account individually in the estimation of esds in distances, angles and torsion angles; correlations between esds in cell parameters are only used when they are defined by crystal symmetry. An approximate (isotropic) treatment of cell esds is used for estimating esds involving l.s. planes.
Refinement. Refinement of F^2^ against ALL reflections. The weighted R-factor wR and goodness of fit S are based on F^2^, conventional R-factors R are based on F, with F set to zero for negative F^2^. The threshold expression of F^2^ > 2sigma(F^2^) is used only for calculating R-factors(gt) etc. and is not relevant to the choice of reflections for refinement. R-factors based on F^2^ are statistically about twice as large as those based on F, and R- factors based on ALL data will be even larger.

### Fractional atomic coordinates and isotropic or equivalent isotropic displacement parameters (Å^2^)

**Table:**

	*x*	*y*	*z*	*U*_iso_*/*U*_eq_	
Nd1	0.5000	0.51140 (3)	0.2500	0.0166 (3)	
Cl2	0.0000	0.55946 (19)	0.7500	0.0334 (8)	
O4	0.6995 (7)	0.4591 (3)	0.1133 (5)	0.0263 (13)	
H8	0.802 (4)	0.448 (5)	0.131 (7)	0.032*	
H9	0.694 (9)	0.459 (5)	0.020 (2)	0.032*	
O1	0.5450 (7)	0.3957 (3)	0.5981 (6)	0.0250 (13)	
O2	0.6051 (7)	0.4301 (3)	0.3977 (5)	0.0225 (12)	
O3	0.2471 (7)	0.5501 (3)	0.1620 (6)	0.0242 (13)	
H6	0.163 (6)	0.526 (3)	0.195 (10)	0.029*	
H7	0.211 (8)	0.5964 (15)	0.157 (9)	0.029*	
C6	0.5817 (8)	0.3855 (4)	0.4827 (8)	0.0164 (15)	
C3	0.6021 (10)	0.3129 (4)	0.4415 (8)	0.0217 (17)	
C2	0.6744 (10)	0.2956 (4)	0.3247 (8)	0.0261 (19)	
H2	0.7100	0.3295	0.2691	0.031*	
C4	0.5480 (12)	0.2591 (4)	0.5189 (10)	0.030 (2)	
H4	0.4971	0.2678	0.5970	0.035*	
N1	0.6459 (9)	0.1776 (4)	0.3683 (7)	0.0286 (17)	
C5	0.5713 (12)	0.1933 (4)	0.4775 (9)	0.030 (2)	
H5	0.5329	0.1581	0.5285	0.036*	
C1	0.6936 (11)	0.2279 (4)	0.2911 (10)	0.030 (2)	
H1	0.7412	0.2173	0.2123	0.036*	

### Atomic displacement parameters (Å^2^)

**Table:**

	*U*^11^	*U*^22^	*U*^33^	*U*^12^	*U*^13^	*U*^23^
Nd1	0.0210 (4)	0.0139 (4)	0.0148 (4)	0.000	−0.0010 (2)	0.000
Cl2	0.0240 (15)	0.0327 (18)	0.043 (2)	0.000	−0.0092 (13)	0.000
O4	0.025 (3)	0.040 (4)	0.014 (3)	0.008 (3)	0.001 (2)	−0.002 (3)
O1	0.034 (3)	0.019 (3)	0.022 (3)	−0.002 (3)	0.007 (3)	−0.005 (2)
O2	0.025 (3)	0.016 (3)	0.026 (3)	−0.001 (2)	−0.006 (3)	0.007 (2)
O3	0.022 (3)	0.015 (3)	0.035 (3)	0.005 (2)	0.005 (3)	0.005 (2)
C6	0.006 (4)	0.018 (4)	0.025 (4)	0.000 (3)	−0.008 (3)	0.000 (3)
C3	0.023 (4)	0.022 (4)	0.021 (4)	0.000 (3)	0.002 (3)	0.001 (3)
C2	0.026 (5)	0.026 (4)	0.027 (4)	−0.003 (4)	0.006 (4)	0.002 (4)
C4	0.037 (5)	0.020 (4)	0.032 (5)	−0.005 (4)	0.003 (4)	0.002 (4)
N1	0.040 (4)	0.021 (4)	0.025 (4)	0.002 (3)	−0.009 (3)	−0.009 (3)
C5	0.040 (6)	0.015 (4)	0.035 (5)	0.000 (4)	0.006 (4)	0.004 (3)
C1	0.035 (5)	0.023 (5)	0.031 (5)	0.005 (4)	−0.003 (4)	−0.008 (4)

### Geometric parameters (Å, °)

**Table:**

Nd1—O1^i^	2.425 (6)	O3—H6	0.95 (2)
Nd1—O1^ii^	2.425 (6)	O3—H7	0.97 (2)
Nd1—O2^iii^	2.385 (5)	C6—C3	1.501 (10)
Nd1—O2	2.385 (5)	C3—C2	1.392 (11)
Nd1—O3^iii^	2.544 (6)	C3—C4	1.404 (12)
Nd1—O3	2.544 (6)	C2—C1	1.385 (12)
Nd1—O4^iii^	2.480 (6)	C2—H2	0.9300
Nd1—O4	2.480 (6)	C4—C5	1.377 (12)
O4—H8	0.96 (2)	C4—H4	0.9300
O4—H9	0.95 (2)	N1—C5	1.330 (12)
O1—C6	1.234 (9)	N1—C1	1.332 (12)
O1—Nd1^i^	2.425 (6)	C5—H5	0.9300
O2—C6	1.248 (9)	C1—H1	0.9300
			
O2^iii^—Nd1—O2	95.7 (3)	Nd1—O4—H8	132 (5)
O2^iii^—Nd1—O1^i^	147.2 (2)	Nd1—O4—H9	121 (5)
O2—Nd1—O1^i^	99.8 (2)	H8—O4—H9	104 (3)
O2^iii^—Nd1—O1^ii^	99.8 (2)	C6—O1—Nd1^i^	140.4 (5)
O2—Nd1—O1^ii^	147.2 (2)	C6—O2—Nd1	147.2 (5)
O1^i^—Nd1—O1^ii^	82.1 (3)	Nd1—O3—H6	115 (5)
O2^iii^—Nd1—O4^iii^	78.0 (2)	Nd1—O3—H7	127 (5)
O2—Nd1—O4^iii^	69.63 (19)	H6—O3—H7	103 (3)
O1^i^—Nd1—O4^iii^	80.7 (2)	O1—C6—O2	125.9 (7)
O1^ii^—Nd1—O4^iii^	141.9 (2)	O1—C6—C3	116.9 (7)
O2^iii^—Nd1—O4	69.63 (19)	O2—C6—C3	117.2 (7)
O2—Nd1—O4	78.0 (2)	C2—C3—C4	116.8 (8)
O1^i^—Nd1—O4	141.9 (2)	C2—C3—C6	121.8 (7)
O1^ii^—Nd1—O4	80.7 (2)	C4—C3—C6	121.4 (7)
O4^iii^—Nd1—O4	131.0 (3)	C1—C2—C3	120.2 (8)
O2^iii^—Nd1—O3^iii^	140.41 (19)	C1—C2—H2	119.9
O2—Nd1—O3^iii^	68.36 (19)	C3—C2—H2	119.9
O1^i^—Nd1—O3^iii^	72.4 (2)	C5—C4—C3	119.1 (9)
O1^ii^—Nd1—O3^iii^	81.4 (2)	C5—C4—H4	120.4
O4^iii^—Nd1—O3^iii^	124.35 (18)	C3—C4—H4	120.4
O4—Nd1—O3^iii^	71.60 (19)	C5—N1—C1	118.5 (7)
O2^iii^—Nd1—O3	68.36 (19)	N1—C5—C4	123.2 (8)
O2—Nd1—O3	140.41 (19)	N1—C5—H5	118.4
O1^i^—Nd1—O3	81.4 (2)	C4—C5—H5	118.4
O1^ii^—Nd1—O3	72.4 (2)	N1—C1—C2	122.0 (9)
O4^iii^—Nd1—O3	71.60 (19)	N1—C1—H1	119.0
O4—Nd1—O3	124.35 (18)	C2—C1—H1	119.0
O3^iii^—Nd1—O3	145.1 (3)		

Symmetry codes: (i) −*x*+1, −*y*+1, −*z*+1; (ii) *x*, −*y*+1, *z*−1/2; (iii) −*x*+1, *y*, −*z*+1/2.

### Hydrogen-bond geometry (Å, °)

**Table:**

*D*—H···*A*	*D*—H	H···*A*	*D*···*A*	*D*—H···*A*
O3—H6···Cl2^iv^	0.95 (6)	2.29 (6)	3.211 (7)	163 (6)
O3—H7···N1^v^	0.97 (4)	1.72 (4)	2.685 (10)	174 (9)
O4—H8···Cl2^i^	0.96 (4)	2.15 (5)	3.041 (6)	155 (6)
O4—H9···O3^vi^	0.95 (2)	1.93 (3)	2.841 (8)	160 (8)

Symmetry codes: (iv) −*x*, −*y*+1, −*z*+1; (v) *x*−1/2, *y*+1/2, −*z*+1/2; (i) −*x*+1, −*y*+1, −*z*+1; (vi) −*x*+1, −*y*+1, −*z*.
